# Construction of Bio-TiO_2_/Algae Complex and Synergetic Mechanism of the Acceleration of Phenol Biodegradation

**DOI:** 10.3390/ma16103882

**Published:** 2023-05-22

**Authors:** Jinxin Guo, Xiaoman Guo, Haiyan Yang, Daohong Zhang, Xiaogeng Jiang

**Affiliations:** 1 Tianjin Key Laboratory of Aquatic Science and Technology, School of Environmental and Municipal Engineering, Tianjin Chengjian University, Jinjing Road 26, Tianjin 300384, China; 2 School of Mechanical Engineering, Tiangong University, Tianjin 300387, China

**Keywords:** microalgae, biosynthesis, TiO_2_, photocatalysis, biodegradation

## Abstract

Microalgae have been widely employed in water pollution treatment since they are eco-friendly and economical. However, the relatively slow treatment rate and low toxic tolerance have seriously limited their utilization in numerous conditions. In light of the problems above, a novel biosynthetic titanium dioxide (bio-TiO_2_ NPs)—microalgae synergetic system (Bio-TiO_2_/Algae complex) has been established and adopted for phenol degradation in the study. The great biocompatibility of bio-TiO_2_ NPs ensured the collaboration with microalgae, improving the phenol degradation rate by 2.27 times compared to that with single microalgae. Remarkably, this system increased the toxicity tolerance of microalgae, represented as promoted extracellular polymeric substances EPS secretion (5.79 times than single algae), and significantly reduced the levels of malondialdehyde and superoxide dismutase. The boosted phenol biodegradation with Bio-TiO_2_/Algae complex may be attributed to the synergetic interaction of bio-TiO_2_ NPs and microalgae, which led to the decreased bandgap, suppressed recombination rate, and accelerated electron transfer (showed as low electron transfer resistance, larger capacitance, and higher exchange current density), resulting in increased light energy utilization rate and photocatalytic rate. The results of the work provide a new understanding of the low-carbon treatment of toxic organic wastewater and lay a foundation for further remediation application.

## 1. Introduction

With the development of industry, phenol has been widely used as one of the raw materials in plastics, oil refineries, paper making, coal processing, and pharmaceutical industries, becoming one of the most common organic pollutants [1,2]. At the same time, the extensive utilization of phenol has resulted in a dramatic increase in phenolic wastewater discharged into natural water bodies. As a biotoxic organic matter, phenol can produce toxic stimulation to living organisms in the environment [3] and has been classified as a major pollutant by the US Environmental Protection Agency (USEPA) and the National Pollutant Release Inventory (NPRI) [4] of Canada. Prevalent studies about phenolic wastewater treatment methods include steam distillation [5], adsorption [6], extraction [7], chemical oxidation [8], and biodegradation [9], which are expensive and require further treatment. Some studies have also employed enzymes and cell systems coupling with nanoparticles [10]. However, the construction of most systems is complicated, while the systems generated by absorption are low efficient owing to a relatively long treatment period [11,12,13] (Table S1). Hence, it is imperative to seek a phenol removal strategy with high efficiency, low cost, and easy operation, which is of great significance to environmental protection and human health.

Microalgae utilize solar energy to fix carbon dioxide [14], called photosynthesis. This approach has been employed as an environmentally friendly and sustainable alternative to the energy-intensive and conventional biological treatment processes prevalently used today [15]. The idea of microalgae for biodegradation has been proposed for the first time by Oswald and Gotaas [16]. Following studies have shown that some cyanobacteria and eukaryotic microalgae, such as *Chlorella* species, *Scenedesmus* species, *Selenastrum capricornutum*, *Tetraselmis marina*, *Ochromonas danica*, *Lyngbya gracilis*, *Nostoc punctiforme*, *Oscillatoria animalis*, and *Phormidium foveolamm*, may be able to transform phenolic compounds through a biological process [17]. By producing valuable by-products (e.g., proteins, lipids), microalgae have shown great potential for environmentally sustainable development. However, the toxicity tolerance of microalgae to harmful pollutants was poor, which affected the treatment efficiency of phenolic wastewater [18]. To increase the treatment efficiency of microalgae-based systems, several strategies have been proposed, including selecting appropriate microalgae strains [18], optimizing the growth conditions [19], and developing integrated systems with other treatment technologies [20].

Photocatalysis is an environmentally friendly method for the degradation and removal of pollutants in wastewater [21]. Researchers kept on synthesizing new photocatalysts, especially for organic contaminants removal [22,23,24,25]. Titanium oxide (TiO_2_) was the most promising photocatalyst because of its environmental friendliness, low cost, and stable properties. Studies have been reported to improve the performance of TiO_2_ by combining it with other materials, such as doping cations (rare earth and transition metals) [26,27] or anions (halogen, sulfur, carbon, and nitrogen) [28], since high photoelectron-hole pair recombination rate reduced the efficiency of TiO_2_. Studies about TiO_2_ with chlorophyll have been reported to broaden TiO_2_ absorbance wavelength to visible light and reduce photoelectron-hole pair recombination rate [29,30]. Due to low solubility in water, in those studies, the chlorophyll was in the form of powder [29] or directly sprayed on leaves [30]. However, research about TiO_2_ with biological organisms is rarely reported due to its poor solubility and toxicity.

In this work, biosynthetic TiO_2_ nanoparticles (bio-TiO_2_ NPs) were composed in microalgae solution to generate a synergetic system, Bio-TiO_2_/Algae complex, in order to solve the problems in microalgae biodegradation, such as slow treatment rate and low toxic tolerance. Compared to chemically synthesized TiO_2_ NPs, the synthesized bio-TiO_2_ NPs were biocompatible with microalgae, which could collaborate with microalgae and enhance phenol degradation rate by 2.27 times. Several parameters were analyzed to understand the synergetic mechanism of the Bio-TiO_2_/Algae complex, including cell metabolism, antioxidant stress, electron transfer rate, and photogenerated electron-hole trapping experiments. The synergetic mechanism of phenol degradation by Bio-TiO_2_/Algae was investigated, which introduced a possible idea for wastewater treatment in conjunction with carbon capture and utilization.

## 2. Materials and Methods

### 2.1. Chemicals and TiO_2_ Biosynthesis

All agents were analytical grade and used without further purification. L-Arginine, L-Cysteine, and Tyzor LA-Lactid acid chelated titanate (Ti-BALDH) were purchased from Macklin (Shanghai, China). Phenol and other chemicals were obtained from Macklin (Shanghai, China). Using Ti-BALDH as raw material, bio-TiO_2_ NPs were prepared by the bionic dehydrating method [31]. Briefly, 0.0053 g L-Arginine powder and 0.01 g L-Cysteine were dissolved in 20 mL deionized water and adjusted to pH 7 with concentrated nitric acid. After stirring for 15 min, 200 μL Ti-BALDH (0.294 wt%) was dropped into the mixture and stirred for another 20 min, forming slight white turbidity. To synthesize bio-TiO_2_/Algae complex, 0.0053 g L-Arginine powder, and 0.01 g L-Cysteine were dissolved in 20 mL *T. obliquus* suspensions and stirred for 15 min. After adding 200 μL Ti-BALDH (0.294 wt%) and stirring for another 20 min, slight white turbidity was formed.

### 2.2. Microalgae Culture and Growth Conditions

*Tetradesmus obliquus* (*T. obliquus*, FACHB-12), *Chlorella vulgaris* (*C. vulgaris*, FACHB-8), and *Chlorella ellipsoidea* (*C. ellipsoidia*, FACHB-40) were purchased from the Institute of Wuhan Hydrobiology, Chinese Academy of Sciences (Wuhan, China), with a density of 1 × 10^6^ algae cells mL^−1^. BG11 was used as a growth medium for microalgae routine culture, which included: NaNO_3_ 1.5 g/L, K_2_HPO_4_ 0.004 g/L, MgSO_4_·7H_2_O 0.075 g/L, CaCl_2_·2H_2_O 0.036 g/L, Citric acid 0.006 g/L, Ferric ammonium citrate 0.006 g/L, EDTANa_2_ 0.001 g/L and Na_2_CO_3_ 0.02 g/L. The trace elements (A5) of the 1 mL/L included: H_3_BO_3_ 2.86 g/L, MnCl_2_·4H_2_O 1.86 g/L, ZnSO_4_·7H_2_O 0.22 g/L, Na_2_MoO_4_·2H_2_O 0.39 g/L, CuSO_4_·5 H_2_O 0.08 g/L and Co(NO_3_)_2_·6 H_2_O 0.05 g/L, pH 7.1. Those three microalgae were screened for phenol degradation without the aid of nanoparticles, and *T. obliquus* was chosen in the following studies.

The inoculum of *T. obliquus* was cultured in triangular vials (100 mL) in a sterile BG11 base medium. Those culture vials were placed in a light incubator (OSRAM DULUX L 36 W, 4500 lux, 30 °C), and the light condition was set to a light/dark cycle of 16/8 h. The inoculum amount of microalgae was 1% (V_inoculum_/V_media_), with an absorbance of 1.0 at a 680 nm optical density (OD 680 nm), equivalent to a microalgae biomass of 0.8222 g L^−1^.

### 2.3. Characterization Methods

The morphology of *T. obliquus* cells and the distribution of TiO_2_ on the cell surface were observed using a TESCAN scanning electron microscope (SEM; Zeiss TESCAN MIRA4, Carl Zeiss AG, Jena, Germany) equipped with an energy dispersive X-ray spectroscopy (EDS; Oxford Xplore 30). Specifically, the samples were fixed in 2.5% glutaraldehyde (6–12 h) and dehydrated with 50%, 70%, 80%, 90%, and 100% anhydrous ethanol in a gradient manner, then immersed in 50% isopentyl acetate (*V*/*V*) and 100% isopentyl acetate for 15 min successively and coated with a layer of Pt on the surface. Then the samples were observed and analyzed by SEM and EDS. Then, those as-prepared powders were scanned by X-ray diffraction patterns (XRD) in the region of 5^◦^ to 90^◦^ using an X-ray diffractometer (Rigaku Ultima IV, Tokyo, Japan) with Cu Kα radiation. The X-ray photoelectron spectroscopy (XPS) study was performed with an Escalab 250XI spectrometer (Thermo Fisher Scientific, Waltham, MA, USA) using calibration C_1s_ at 284.8 eV.

The UV-Vis absorption of both bio-TiO_2_ NPs and Bio-TiO_2_/Algae was scanned with a UV-Vis spectrometer (Agilent, Santa Clara, CA, USA). The distribution of biosynthesized TiO_2_ NPs in the Bio-TiO_2_/Algae synergetic system was observed by High-Resolution Transmission Electron Microscope (HRTEM, HT7800, Hitachi, Tokyo, Japan) on the copper net.

### 2.4. Photocatalytic Degradation of Phenol

Three systems were established in this study, including Algae (*T. obliquus* only), Bio-TiO_2_/Algae (*T. obliquus* + bio-TiO_2_ NPs), and a control system (inactivated *T. obliquus* (60 °C for 20 min) + bio-TiO_2_ NPs). The inoculum of three systems (OD 680 = 1.0, V_inoculum_/V_media_ = 1%) was cultured in 100 mL triangular vials with 60 mL sterile BG11 base medium, followed by the addition of 100 mg L^−1^ phenol. Those culture vials were placed in a light incubator (4500 lux, 30 °C), and the light condition was set to a light/dark cycle of 16/8 h.

For phenol detection, the supernatant was collected by centrifugation at 6000 rpm for 5 min and filtrated with a 0.45 μm organic membrane [21] before testing with a high-performance liquid chromatography (HPLC, LC-1260, Agilent, USA) equipped with a ZORBAX SB-C18 column (150 mm × 4.6 mm × 5 μm). Mobile phase A was ultra-pure water, and mobile phase B was methyl alcohol (A:B = 40:60). The flow rate of the mobile phases was 1.0 mL min^−1^, and the detection wavelength was 270 nm.

### 2.5. Electrochemical Characteristic Experiments

Electrochemical characterization of Bio-TiO_2_/Algae was performed, including cyclic Voltammetry (CV), electrochemical impedance spectroscopy (EIS), and Tafel. Experiments were conducted using an electrochemical workstation (CHI 660E, CH Instrument Inc., Shanghai, China) with a three-electrode chamber [32]. A glassy carbon electrode, platinum wire electrode, and Ag/AgCl electrode were used as working electrode, counter electrode, and reference electrode, respectively. The working electrode was polished with Al_2_O_3_ powder with particle sizes of 5 mm, 1 mm, 0.3 mm, and 0.05 mm successively. Then, the working electrode was ultrasonically cleaned with deionized water, anhydrous ethanol, and deionized water for 30 s each. Finally, the working electrode was tested with 5 mM potassium ferricyanide as a solute and 0.1 M potassium chloride as a solvent. CV was performed at a voltage of −1.5–1.5 V and a scan rate of 50 mV/s. EIS was performed at 1–10^6^ Hz frequency, and the equivalent circuit of EIS data was simulated by Zview 2.0 software. The Tafel curve was performed at a voltage of −1.5–1.5 V and a scan rate of 10 mV/s.

### 2.6. Free Radical Capture Experiments

The roles of photogenerated electrons (e^−^), photoholes (h^+^), hydroxyl radicals (·OH), and O^2−^ in the catalytic degradation of phenolic pollutants in the system were determined by trapping experiments with trapping agents. The trapping agents used included Potassium bromate, ethylenediamine tetraacetic acid disodium salt (EDTA-2Na), tert-butanol (t-BuOH), and 4-hydroxy-2,2,6,6-tetramethyl-1-piperidinylox (TEMPO). The steps were similar to phenol degradation experiments. After the addition of phenol, a final concentration of 0.5 mM or 5 mM trapping agents was added to the culture vials. The phenol concentration was detected till 52 h.

## 3. Results and Discussion

### 3.1. Synthesis and Characterization of bio-TiO_2_ NPs and Bio-TiO_2_/Algae Complex

**Figure 1:**
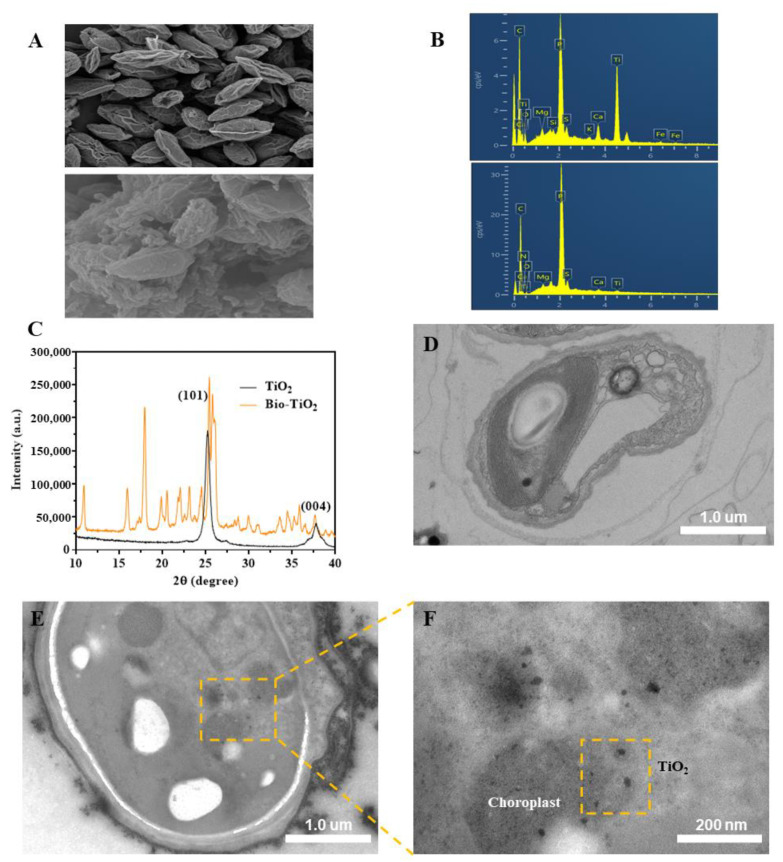
(**A**) SEM image of Algae (**up**) and Bio-TiO_2_/Algae complex (**down**); (**B**) EDS analysis of Bio-TiO_2_/Algae before (**up**) and after (**down**) phenol degradation; (**C**) XRD patterns of chemical synthesized TiO_2_ and bio-TiO_2_; (**D**) HRTEM image of Algae; (**E**) HRTEM image of Bio-TiO_2_/Algae; (**F**) Enlarge of selected region and the black dots are bio-TiO_2_ NPs inside microalgae cell.

The synthesis of bio-TiO_2_ NPs was performed at room temperature. After stirring for 35 min, a slight white precipitate was formed, preliminarily inferring the successful synthesis of the nanoparticles. Compared to chemically synthesized TiO_2_ NPs, the bio-TiO_2_ NPs showed great biocompatibility and could collaborate with microalgae, which was further confirmed by following characterization methods. The SEM images (Figure 1A) indicated that the *T. obliquus* were fusiform with small ends and a large middle and nanoparticles clustered on the surface of microalgae cells. The elemental composition was analyzed by EDS to confirm the presence of Ti in nanoparticles on the cell surface. The EDS (Figure 1B) analysis at the beginning of phases I and the end of phase III of the experiment showed 18.96 and 0.67 wt% of elemental Ti, respectively. The significantly decreased Ti content may be due to shedding during the photocatalytic process [21] or the increased biomass (leading to a relative decrease in Ti content). The attachment of bio-TiO_2_ NPs to microalgae was verified by XRD and XPS. XRD spectrum (Figure 1C) analyzed the crystal structure of the pre-prepared sample appeared 2θ = 25.3 and 37.8, 25.3 anatase characteristic peaks, indicating that the diffraction peaks of the bio-TiO_2_ NPs were similar to those of anatase type nanoparticles [33]. However, the bio-TiO_2_ NPs had more diffraction peaks at more positions, which might be attributed to biosynthesis containing more impurities, including 2θ = 17.94 (possibly reflected in the L-Arginine [34] and L-Cysteine [35]) and 26.10 (possibly indicated to the L-Cysteine [35]).

The XPS was used to further study the element composition and Ti valence state of the pre-prepared sample (Figure S1). The presence of Ti, O, C, and N elements in the sample was observed in Figure S1A, which was consistent with the EDS analysis result. Peaks at 458.0 and 463.8 eV should be ascribed to Ti 2p_3/2_ and Ti 2p_1/2_ and demonstrate the existence of the Ti^4+^ state. The oxygen in the sample could be divided into the lattice oxygen (O_Ti-O-Ti_, 530.1 eV) and the surface hydroxyl oxygen (O_Ti-O-H_, 531.4 and 531.5 eV). The presence of O_Ti-O-Ti_ and Ti^4+^ confirmed the successful synthesis of TiO_2_ NPs. In the high-resolution XPS diagram of C 1s of the sample, 284.4, 285.1, and 288.0 eV were observed, corresponding to C-C, C=O, and O-C=O bonding, respectively. The N 1s core peak was observed at 400.4 eV, which corresponded to Ti-N bonding. The C 1s and N 1s indicated that the synthesized bio-TiO_2_ NPs were coupled with amino acids.

In HRTEM images (Figure 1D–F), the bio-TiO_2_ NPs surrounded microalgae (Figure 1E), and some of them passed cell membranes and got close to the chloroplast (CLP) (Figure 1F). The absorbance wavelength of the Bio-TiO_2_/Algae complex shifted to visible light (Figure S2), also indicating the interaction of CLP in microalgae and bio-TiO_2_ NPs, since electron transfer from TiO_2_ NPs to CLP resulted in a narrower bandgap. The two bandgaps were calculated to be 3.10 eV for bio-TiO_2_/CLP and 3.24 eV for bio-TiO_2_ with empirical formula (Eg = 1240/λg) [36].

In conclusion, the bio-TiO_2_ NPs were successfully synthesized and coupled with microalgae, the Bio-TiO_2_/Algae complex, which provided a basis for the synergetic biodegradation of phenol.

### 3.2. Phenol Degradation Promotion by Bio-TiO_2_/Algae Complex

**Figure 2:**
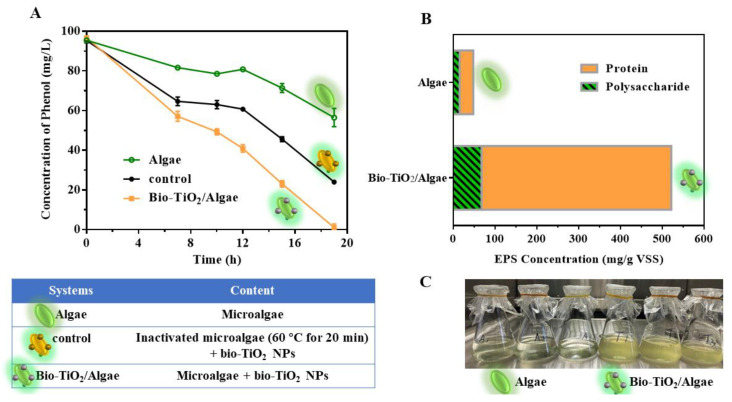
(**A**) Phenol degradation results with Algae, Control, and Bio-TiO_2_/Algae systems; (**B**) Microalgae EPS expression of Algae and Bio-TiO_2_/Algae during phenol degradation; (**C**) Images of Algae and Bio-TiO_2_/Algae during phenol degradation (three replicates were performed).

The effect of Bio-TiO_2_/Algae on phenol degradation was tested. The photocatalytic degradation efficiency of phenol by the Bio-TiO_2_/Algae, Algae, and control system are shown in Figure 2A. The phenol degradation efficiency of Bio-TiO_2_/Algae reached 98.95 ± 1.77% at 19 h, which enhanced 2.27 times and 1.32 times compared with that of the Algae system (40.88 ± 4.62% at 19 h) and control system (74.89 ± 1.12% at 19 h). The degradation rate of phenol in the control system was 1.75 times that of the Algae system. Bio-TiO_2_/Algae complex showed the fastest phenol degradation rate, followed by the control (photocatalysis only) and Algae (respiration only) system. Therefore, the contribution of the bio-TiO_2_ NPs photocatalytic degradation was greater than that of microalgae biodegradation. Meanwhile, phenol was degraded via photocatalysis of bio-TiO_2_ NPs to produce non-toxic, low toxic, and easily biodegradable intermediate metabolites [37,38]. Moderated toxicity to microalgae could be observed from the improved CLP content of microalgae cells than that in the Algae system (Figure 1C), which was consistent with previous reports [30]. Meanwhile, visible light absorption wavelength and narrower bandgap of the TiO_2_/CLP complex resulted in enhanced electrons transfer from TiO_2_ to CLP [30], which improved the light energy utilization rate and photosynthetic efficiency (Figure 2C), thereby enhancing the degradation ability of phenol.

### 3.3. Metabolic Activity Analysis of Microalgae in Bio-TiO_2_/Algae Complex

#### 3.3.1. EPS Excretion in Bio-TiO_2_/Algae Complex

EPS has been reported as positively correlated with biological activity since more active cells can secret more extracellular polymer [32,39]. In this study, the bio-TiO_2_ NPs increased the CLP amount and photosynthetic efficiency of microalgae, which may also impact EPS secretions. Therefore, EPS secretion of Bio-TiO_2_/Algae and Algae system were explored. As shown in Figure 2B, polysaccharide (PS) and protein (PN) are the main components of EPS, and the sum of PS and PN is defined as the total EPS content [40]. The total amount of EPS secreted by *T. obliquus* was 521.86 mg/g VSS and 48.12 mg/g VSS in the Bio-TiO_2_/Algae and Algae system at 19 h when phenol degradation was completed with Bio-TiO_2_/Algae. The PS level was 68.12 mg/g VSS in Bio-TiO_2_/Algae, which was lower in the Algae system (15.85 mg/g VSS). The PN level was higher in Bio-TiO_2_/Algae (453.74 mg/g VSS) compared to the Algae system (32.27 mg/g VSS). Therefore, the addition of TiO_2_ NPs largely increased the EPS level (by 1084.50%), especially PN secretion (by 1406.07%). It has been reported that the loosely bound EPS (LB-EPS) contains almost no PN, while the tightly bound-EPS (TB-EPS) are composed of PN and PS in variable ratios [41]. Therefore, the significantly increased PN in extracted EPS indicated a raised level of excreted TB-EPS, which could combine with the bio-TiO_2_ NPs better because of stronger and less reversible adsorption on solid surfaces [39]. In addition, it has been reported that higher levels of PN in EPS were closely related to biological activity [42] since PN is a major component of organisms and most enzymes [39]. Bio-TiO_2_/Algae showed the fastest degradation rate of phenol, which might be attributed to the higher content of PN in this system. Increased EPS secretion indicated that the bio-TiO_2_ NPs enhanced the metabolic activity of *T. obliquus* and improved the stress response to the surrounding environment, which was further verified by the following tests.

#### 3.3.2. Analysis of ETSA in Bio-TiO_2_/Algae Complex

**Figure 3:**
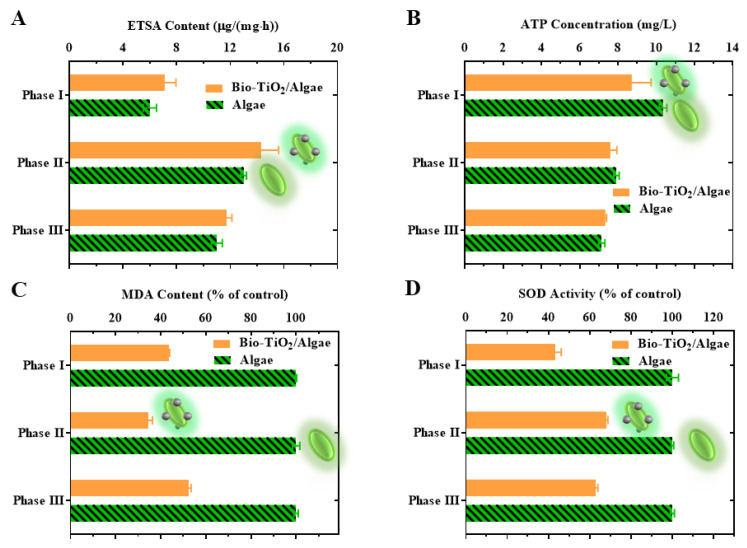
MDA (**A**), SOD (**B**), ETSA (**C**), and ATP (**D**) contents of Bio-TiO_2_/Algae and Algae during phenol degradation.

A high ETSA value can reflect the high respiratory activity of microorganisms and high electron transfer efficiency [43]. ETSA was usually determined with INT, which acted as an electron acceptor to measure the respiratory dehydrogenase activity of heterotrophic microorganisms [32]. The ETSA value increased from phase I to phase II and decreased in phase III for both systems (Figure 3C). The raised ETSA value in phase II might be due to the increase in biomass and substrate abundance, while the dropped ETSA value in phase III was due to a shortage of substrate. The ETSA content was slightly higher in the Bio-TiO_2_/Algae complex, which was consistent with the increased degradation rate of phenol.

#### 3.3.3. Measurement of ATP Level in Bio-TiO_2_/Algae Complex

ATP provided energy for microorganisms, indicated the utilization of organic matter in TCA [44], and was also an energy source in the carbon fixation stage of photosynthesis, thus promoting the Calvin cycle [45]. As shown in Figure 3D, the ATP value in phase I, II, and III of Bio-TiO_2_/Algae were 8.71 ± 1.02, 7.59 ± 0.35, and 7.33 ± 0.07 g/(mg·h), which were 10.35 ± 0.20, 7.92 ± 0.14 and 7.15 ± 0.16 g/(mg·h) of Algae system. ATP levels in both Bio-TiO_2_/Algae and Algae systems decreased during the reaction, but the decrease was much faster in the Algae system. The reduced ATP may be due to long-term exposure to aqueous phenol solution, which inhibited the photosynthesis of microalgae and reduced the electron transfer chain. At the same time, it has been reported that interaction between TiO_2_ and CLP could enhance the formation of ATP [30]. The change in ATP level indicated that the bio-TiO_2_ NPs played a positive role in microalgae metabolism [46] and were able to elevate energy levels to a certain extent to improve the degradation efficiency of phenol.

### 3.4. Stress Response Analysis of Microalgae in Bio-TiO_2_/Algae Complex

Though improved CLP content of microalgae was observed during phenol degradation, nanoparticle toxicity to microalgae was still under concern. Therefore, stress response tests were performed on microalgae cells in Bio-TiO_2_/Algae and Algae system. Levels of malondialdehyde (MDA) and superoxide dismutase (SOD) in the Bio-TiO_2_/Algae and Algae system were tested at the end of phase I, II, and III during phenol degradation. Figure 3A showed that lipid peroxides (MDA), a membrane damage metabolite, in the Algae system were similar during the whole process. However, the MDA level of Bio-TiO_2_/Algae decreased from phase I to phase II but increased in phase III, which was 43.67 ± 0.97%, 34.71 ± 1.64%, and 52.51 ± 0.26% in phase I, phase II, and phase III, compared to MDA level of Algae system. The decreased MDA might be attributed to the promotion of EPS secretion triggered by bio-TiO_2_ NPs, protecting cells from damage [47]. While in stage III, the antioxidant system of microalgae was damaged by oxidative stress due to continuous exposure to adverse environments, resulting in lipid peroxidation of cells and increased MDA content [48]. Therefore, the bio-TiO_2_ NPs enhanced the tolerance of microalgae to toxicity by promoting EPS secretion and reduced lipid peroxidation.

As oxygenic photosynthetic organisms, reactive oxygen species (ROS) were inevitably generated by microalgae when the excitation of photosynthetic pigments exceeded the metabolic demand [49]. ROS could cause oxidative damage to cells and affect photosynthesis. Since SOD is able to resist and remove ROS, its activities can reflect ROS toxicity [50], which was monitored during the whole degradation reaction. Similar to the MDA, the SOD level in the Algae system stayed stable during the reaction (Figure 3B). However, the SOD level of Bio-TiO_2_/Algae increased from phase I to phase II but decreased in phase III, which was 43.25 ± 0.97%, 68.17 ± 0.65%, and 62.94 ± 2.94% in phase I, phase II, and phase III, compared to SOD level of Algae system. The significantly reduced SOD level might be attributed to the presence of bio-TiO_2_ NPs, which photocatalyzed phenol (a toxic substance) and accelerated photosynthesis (by increased electron transfer rate to CLP) [30].

### 3.5. Variations of Extracellular Electron Transfer Behaviors in Bio-TiO_2_/Algae Complex

**Figure 4:**
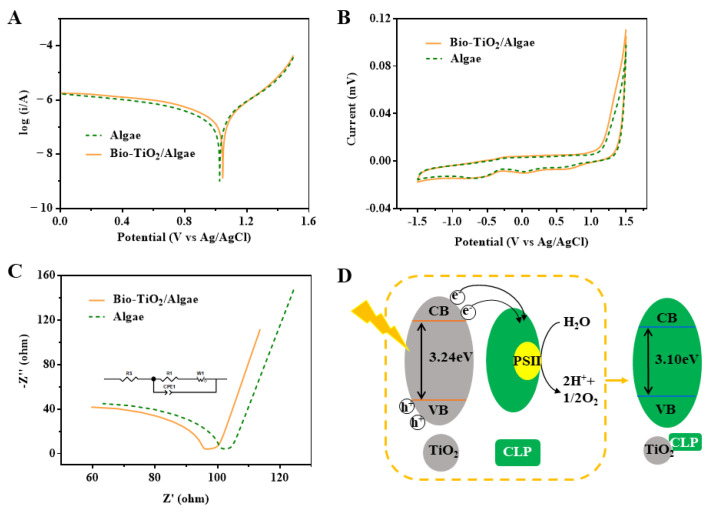
Tafel (**A**), CV (**B**), and IMP (**C**) plots of Bio-TiO_2_/Algae and Algae in phenol degradation; (**D**) Scheme of electron transfer from TiO_2_ to CLP and suppression of electron-hole pair recombination.

Higher electron transfer efficiency was usually observed in improved degradation efficiency. Electrochemical measurement was an important method for evaluating electron transport rate and electrochemical activity in a system [43]. In order to investigate the electrochemical properties of the photocatalytic degradation system, Tafel, CV, and EIS tests were conducted in both Bio-TiO_2_/Algae and Algae systems. As shown in Figure 4A, the exchange current density of Bio-TiO_2_/Algae and Algae systems was 1.455 × 10^−7^ A/cm^2^ and 1.096 × 10^−7^ A/cm^2^, respectively. The higher exchange current density of Bio-TiO_2_/Algae indicated the increased reduction rate [32,51], which could also be reflected in the CV curve (Figure 4B). The capacitor of Bio-TiO_2_/Algae (278.27 μF) was much higher than that of the Algae system (225.51 μF). Tafel test was performed to further verify the improved electron transfer in Bio-TiO_2_/Algae. As shown in Figure 4C, the electronic transfer resistance of the Bio-TiO_2_/Algae and Algae system was 18.06 Ω and 21.25 Ω, respectively. Meanwhile, the SEMI-arc radius of Bio-TiO_2_/Algae was smaller, indicating a lower electron transfer barrier and increased electrical conductivity of phenol photocatalytic degradation in that system [52,53]. The accelerated electron transfer might be attributed to the interaction of bio-TiO_2_ NPs and CLP (Figure 4D). Previous studies also reported that photoelectrons transferred to CLP and suppressed photoelectron-hole pair recombination rate [29,30].

### 3.6. Effect of Photoelectron and Photohole on the Degradation of Phenol by Bio-TiO_2_/Algae Complex

**Figure 5:**
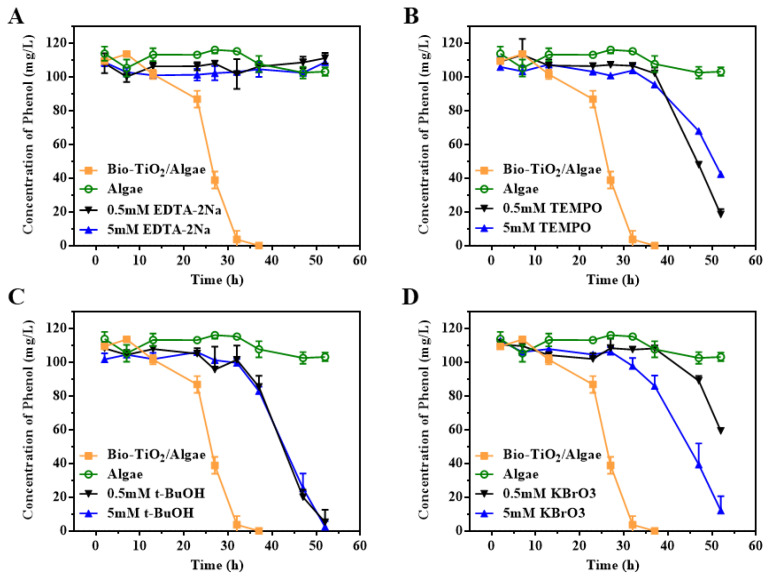
Free radical capture results of (**A**) photogenerated electrons (e^−^), (**B**) photoholes (h^+^), (**C**) hydroxyl radicals (·OH), and (**D**) O^2−^ in Phenol degradation with Bio-TiO_2_/Algae.

According to the literature, the main active species of TiO_2_ were holes during the photocatalysis process [54]. In order to investigate the photocatalytic degradation of phenol by the bio-TiO_2_ NPs and the main substances in the photocatalytic process in Bio-TiO_2_/Algae, the free radical trapping experiment was designed and carried out (Figure 5). EDTA-2Na, TEMPO, t-BuOH, and KBrO_3_ were used to capture h^+^, O_2_^−^, ·OH, and e^−^, respectively [21,55].

When the concentration of trapping agent EDTA-2Na, TEMPO, t-BuOH, and KBrO_3_ was 0.5 mM, the degradation percentage of phenol was 3.46%, 83.95%, 95.48%, and 49.38% at 52 h, respectively. Phenol could hardly be degraded in the presence of an h^+^ trapping agent, indicating that photocatalytic degradation of phenol was mainly caused by h^+^ generated by the photocatalytic and photosynthetic reaction. The consumption of h^+^ was also confirmed by increased pH (Figure S2). When the transfer of e^−^ was blocked by KBrO_3_, interestingly, higher blocker concentration resulted in a faster degradation rate, though the rate was still lower than that of the system without a blocker. Therefore, the consumption of e^−^ would increase the reaction rate. Trapping of O_2_^−^ and ·OH would slow down the degradation rate of phenol, and the rate was similar with both blocker concentrations. Thus, those two free radicals were useful but not critical in the phenol degradation process, and a blocker of 0.5 mM was already enough to block them. Those were consistent with previous literature reports that biodegradation and indirect reactions with photochemically produced hydroxyl radicals and peroxyl radicals, which were expected to be important intermediate products [3].

### 3.7. Synergetic Mechanism of Phenol Degradation by Bio-TiO_2_/Algae Complex

**Figure 6:**
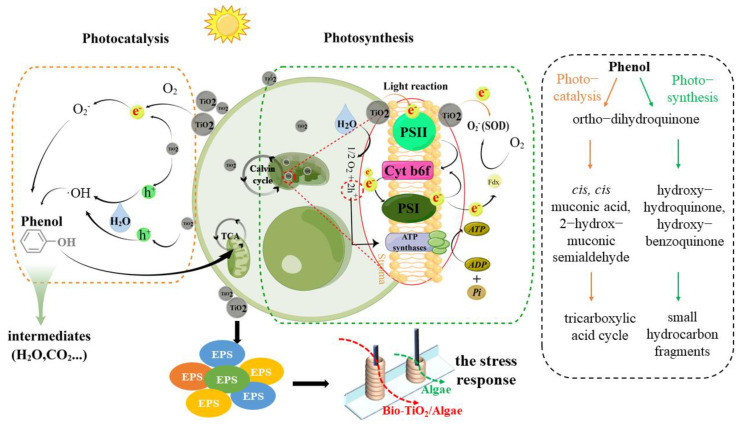
Schematic diagram of the synergetic mechanism of Bio-TiO_2_/Algae in phenol degradation and the possible pathway of degradation process [56,57,58]. (The figure was mainly drawn by Figdraw).

According to previous results, the synergetic mechanism of phenol degradation by Bio-TiO_2_/Algae was proposed (Figure 6). First, the bio-TiO_2_ NPs degraded phenol to non-toxic or low-toxic intermediate metabolites and alleviated the stress of phenol on microalgae cells, resulting in the disinhibition of microalgae cell metabolism and photosynthesis. Second, the bio-TiO_2_ NPs also stimulated EPS excretion of microalgae cells and decreased the oxidative stress and lipid peroxidation damage of cells (represented as low SOD and MDA levels), resulting in increased tolerance of microalgae cells to adverse environments. Meanwhile, the bio-TiO_2_ NPs passed through cell membranes and interacted with CLP, which enhanced the formation of ATP [30]. Thus, the cell metabolic activity and photosynthetic rate were improved, which was indicated as a high ETSA value. A similar result was reported previously, that exogenous nanomaterials were found to be a more direct and easier way to stimulate and regulate cell metabolism, thus enhancing metabolic activity and improving photosynthetic rate [48].

Phenol was degraded by both photocatalysis of bio-TiO_2_ NPs and the tricarboxylic acid cycle of microalgae. Phenol degradation pathway by bio-organism and nanoparticles has been investigated throughout previous studies, shown in Figure 6 [56,57,58,59]. Photocatalytic degradation of phenol was mainly caused by h^+^ generated by the photocatalytic and photosynthetic reactions. Because of the interaction of bio-TiO_2_ NPs and CLP, during the phenol degradation process, e^−^ were rapidly transferred to CLP (Figure 4D) or O_2_, reducing the photoelectron-hole pair recombination rate, supporting more h^+^, and improving the photocatalytic performance of the bio-TiO_2_ NPs. The accelerated electron transfer was shown as low electron transfer resistance, larger capacitance, and higher exchange current density, resulting in increased light energy utilization rate and photocatalytic rate. Meanwhile, O_2_ generated by photosynthesis increased the consumption of e^−^, which further improved photocatalytic efficiency.

Thus, the synergetic reaction of both the bio-TiO_2_ NPs (photocatalysis) and microalgae cells (especially photosynthesis) was formed, which resulted in accelerated biodegradation of phenol.

## 4. Conclusions

In this study, bio-TiO_2_ NPs were successfully synthesized and coupled with microalgae, providing a basis for synergetic biodegradation of phenol as the Bio-TiO_2_/Algae complex, of which the phenol degradation efficiency reached 98.95 ± 1.77% at 19 h, enhancing 2.27 times and 1.32 times compared with that of Algae system (40.88 ± 4.62% at 19 h) and control system (74.89 ± 1.12% at 19 h), respectively.

Bio-TiO_2_/Algae showed the fastest degradation rate of phenol, which might be attributed to the higher content of PN in this system. Increased EPS secretion indicated that the bio-TiO_2_ NPs enhanced the metabolic activity of *T. obliquus* and improved the stress response to the surrounding environment, which was further verified by the ETSA and ATP tests. Electrochemical studies, including Tafel, CV, and EIS tests, verified the increased electron transfer, leading to accelerated degradation of phenol.

The results of the free radical trapping experiment showed that phenol could hardly be degraded in the presence of an h^+^ trapping agent, indicating that photocatalytic degradation of phenol was mainly caused by h^+^ generated by the photo-catalytic and photosynthetic reaction.

Thus, the synergetic reaction of both the bio-TiO_2_ NPs (photocatalysis) and microalgae cells (especially photosynthesis) was formed, which resulted in accelerated biodegradation of phenol.

This study investigated a green and sustainable method for phenolic wastewater treatment. The idea was enlightened for other toxicity low-carbon remediation in natural water.

## Data Availability

The data presented in this study are available upon request from the corresponding author.

## Supplementary Materials

The following supporting information can be downloaded at: https://www.mdpi.com/article/10.3390/ma16103882/s1, Table S1: Comparison of existing systems and Bio-TiO_2_/Algae complex. Table S2: Zeta potential of Algae and Bio-TiO_2_/Algae complex before and after phenol degradation. Figure S1: XPS analysis results of bio-TiO_2_ NPs. Figure S2: UV-Vis absorbance of bio-TiO_2_ and Bio-TiO_2_/Algae. Figure S3: pH change of Bio-TiO_2_/Algae during and after phenol degradation. Figure S4: Efficiency (A) and the kinetic curves (B–D) of phenol degradation with Bio-TiO_2_/Algae complex. References [60,61,62,63,64,65] are cited in the supplementary materials.
