# Supplementary material for: Construction of Bio-TiO2/Algae Complex and Synergetic Mechanism of the Acceleration of Phenol Biodegradation
Source: Materials (Basel). 2023 May 22;16(10):3882. doi: 10.3390/ma16103882 (PMC10221352; doi:10.3390/ma16103882)
Supplement: Supplementary file 1 [file materials-16-03882-s001.zip › materials-2389492-supplementary.pdf]

Supporting Information

Contents

Materials and Methods S2-S3
Supplementary Tables S1-S2 S4-S5
Supplementary Figures S1-S34 S6-S9
Supplementary Discussion S10

Materials and Methods

EPS extraction

Influence of bio-TiO_2 NPs on EPS secretion of microalgae during photocatalytic degradation of phenol was investigated. EPS were extracted from *T. obliquus* by heat extraction [42, 60-62]. The cells were harvested by centrifugation (8000 rpm,10 min) and the cell pellets were washed with 0.9% NaCl (w/v) solution three times, followed by resuspension in 0.9% NaCl (w/v) solution. Protein and polysaccharide contents in EPS were determined using the Coomassie Brilliant Blue G-250 method (with bovine serum albumin (BSA) as a standard) and the anthrone sulfate method).

Detection of MDA and SOD

MDA amount can be detected in following steps. A 10 mL aliquot of *T. obliquus* cells was centrifuged for 10 min at 6000 rpm. The pellet was washed two times wash with PBS. After dissolved in 10 mL PBS, the solution was centrifuged for 10 min at 6000 rpm. The pellet was dissolved in 2 mL riboflavin (20 μmol/L), following by addition of 1 mL nitroblue tetrazolium (1.6 mmol/L), 1 mL Triton X-100 (10%), and 1 mL $NH_2OH\cdot HCl$ (100 mmol/L). The mixture was reacted for 10 min and turned into purple color. After centrifuging for 5 min at 8000 rpm, the absorbance at 560 nm was measured.

MDA concentration was detected as follows. A 10 mL aliquot of *T. obliquus* cells was centrifuged for 10 min at 6000 rpm. The pellet was washed two times wash with PBS and dissolved in 10 mL PBS. Then, a 1.9 mL aliquot of Na_3PO_4 (0.1 mol/L) was added into the resulted solution, and the mixture was incubated for 1 hour (4500 Lux, 30 °C). After addition of 1 mL $C_2HCl_3O_2$ (5%, w/v), the mixture was centrifuged for 25 min at 2300 ×g . Absorbance at 532 nm of the supernatant was measured.

Measurement of ETSA and ATP

Microalgae cell metabolism were studied by testing ETSA and ATP in both systems. A 10 mL aliquot of *T. obliquus* cells was centrifuged for 10 min at 6000 rpm. The pellet was washed two times wash with PBS and dissolved in 10 mL PBS. After addition of 1 mL iodonitrotetrazolium solution (0.2%, w/v), the mixture was incubated in dark for 30 min (141 rpm, 35 °C). The reaction was aborted with 1 mL formaldehyde (37%, w/v), and pellet was collected by centrifuging for 5 min at 6000 rpm. After suspension in 5 mL methanol (V), the solution was incubated for 10 min (141 rpm, 35 °C), following by centrifuging for 10 min at 8000 rpm. Absorbance of the supernatant at 485 nm (D_{485}) and dry weight of pellet (W) were measured. The ETSA amount was calculated as follows:

$$\mathrm{ETSA} = \frac{\mathrm{D}_{485}\mathrm{V}}{kWt}$$

where k refers to slope of ETSA standard curve, which is 0.0547 mL/μg; t is the cultivation time of microalgae.

ATP level of the microalgae were detected in following steps. A 10 mL aliquot of *T. obliquus* cells was centrifuged for 10 min at 6000 rpm. The pellet was resuspended in 6 mL Tris-EDTA buffer (pH 7.0). After boiling for 3 min with water bath, the solution was cooled down to room temperature and passed through a 0.22 μm filter. ATP concentration was detected with HPLC (LC-1260, Agilent, USA) equipped with a ZORBAXSB-C18 column (150 mm × 4.6 mm × 5 μm). Mobile phase A was ammonium acetate (20 mM, pH=4.5) and mobile phase B was acetonitrile

(A:B = 95:5). The flow rate of the mobile phases was 1.0 mL min^{-1} and the detection wavelength was 260 nm.

Supplementary Tables

Constructed system	Utility	Efficiency	Reference
Pseudomonas putida entrapped in Ca-alginate gel beads	Phenol degradation	Phenol (50-600 mg L^{-1}) degraded completely within 48 h	[63]
Pseudomonas putida-sepiolite adsorbed on Zeolite-activated sepiolite-imidazole	Phenol degradation	Phenol (20 mg L^{-1}) degraded completely within 24 h	[13]
Pseudomonas aeruginosa adsorbed on polyurethane foam	Phenol degradation	Phenol (1500 mg L^{-1}) degraded within 750 min	[12]
Nano-TiO_2 and microalgae	Humic acid utilization from piggery biogas slurry	Humic acid removal efficiency of 50.14%, NH_4-N removal efficiency of 84.78%, and TP removal efficiency of 69.58% after 48 hours	[64]

Table S1. Comparison of existing systems and Bio-TiO_2/Algae complex

Zeta potential (mV)	Algae	Bio-TiO_2/Algae
Before reaction	-12.4±0.12	-16.8±0.224
After reaction	-15.3±1.334	-17.6±0.627

Table S2. Zeta potential of Algae and Bio-TiO_2/Algae complex before and after phenol degradation.

Supplementary Figures

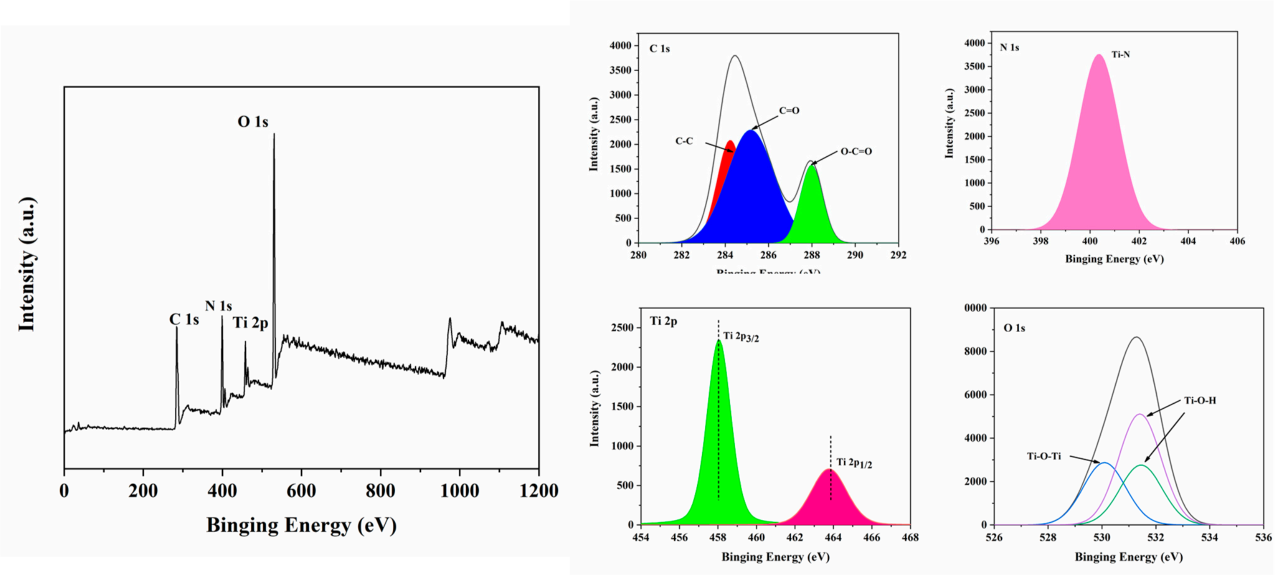

Figure S1. XPS analysis results of bio-TiO_2 NPs

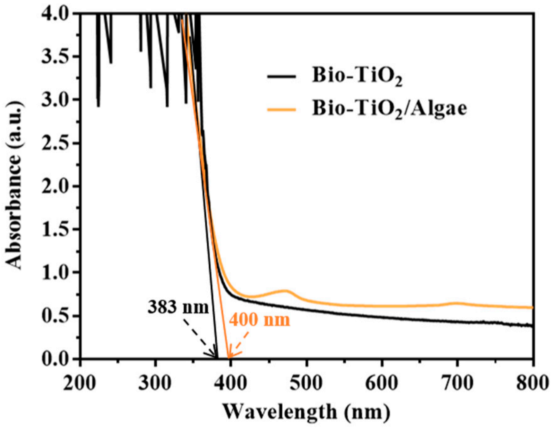

Figure S2. UV-Vis absorbance of bio-TiO_2 and Bio-TiO_2/Algae

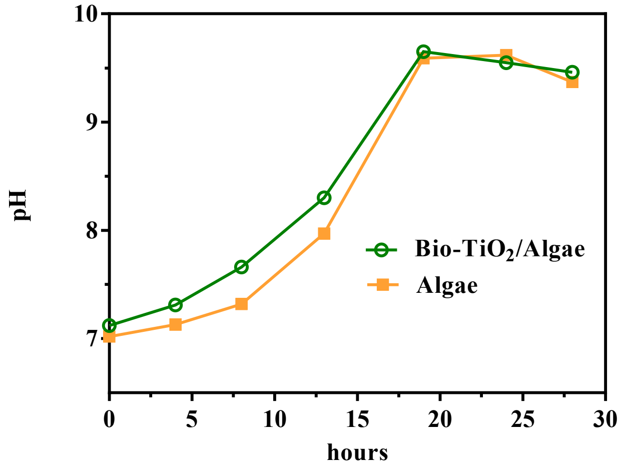

Figure S3. pH change of Bio-TiO_2/Algae during and after phenol degradation

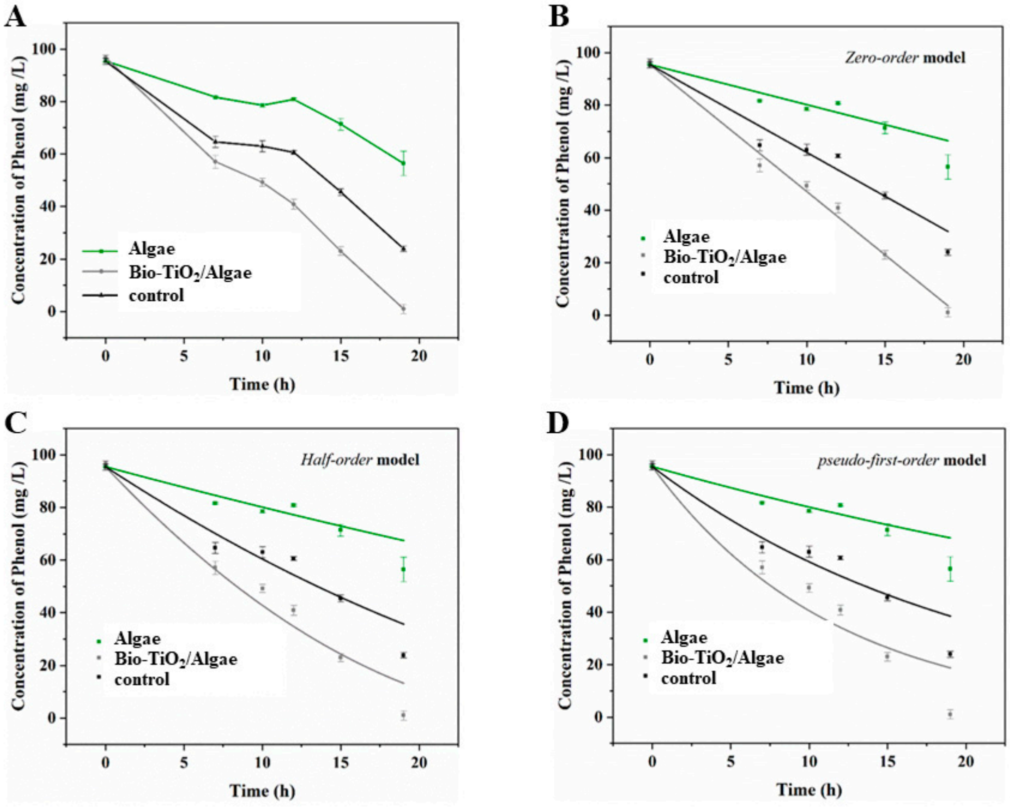

Figure S4. Efficiency (A) and the kinetic curves (B-D) of phenol degradation with Bio-TiO_2/Algae complex

Supplementary Discussion

Phenol degradation promotion by Bio-TiO_2/Algae

The Haldane model was adequate to reflect the phenol for relatively low initial phenol concentrations. However, for higher concentration of phenol, there was a significant difference between the measured value and the estimated value [9]. Typically, the photocatalytic degradation reaction was fitted to the pseudo-first-order reaction kinetics [65]. The photocatalytic degradation of phenol by the Bio-TiO_2/Algae, Algae, and control system were fitted with pseudo-first-order, half-order, and zero-order reaction kinetics (Figure S4). Based on R^2 (kinetics constant) values of the three systems, the pseudo-first-order reaction kinetics was most consistent with phenol degradation reaction by Bio-TiO_2/Algae, and the R^2 of the fitting curve was 0.97096. The calculation was as follows:

$$\frac{C_t}{C_0} = e^{-kt}$$

where t refers to the reaction time, C_0 (mg/L) is the initial concentration of Phenol, C (mg/L) is the concentration of the pollutant at time t, and k is the rate constant.

The photocatalytic degradation rate of phenol was increased by Bio-TiO_2/Algae and Algae system, which was consistent with the prediction of morphology and structure analysis.
